# Nonintubated versus Intubated Lung Volume Reduction Surgery in Patients with End-Stage Lung Emphysema and Hypercapnia

**DOI:** 10.3390/jcm12113750

**Published:** 2023-05-29

**Authors:** Ali Akil, Stephanie Rehers, Stephan Ziegeler, Erik Ernst, Jan Haselmann, Nicolas Johannes Dickgreber, Stefan Fischer

**Affiliations:** 1 Department of Thoracic Surgery and Lung Support, Ibbenbueren General Hospital, 49477 Ibbenbueren, Germany; dr.ali.akil.11@gmail.com (A.A.);; 2 Department of Anesthesiology, Intensive Care Medicine and Pain Management, Ibbenbueren General Hospital, 49477 Ibbenbueren, Germany; 3 Department of Respiratory Medicine and Pulmonary Rehabilitation, Karl-Hansen-Hospital, 33175 Bad Lippspringe, Germany; 4 Department of Respiratory Medicine and Thoracic Oncology, Ibbenbueren General Hospital, 49477 Ibbenbueren, Germany

**Keywords:** LVRS, nonintubated, lung emphysema, hypercapnia, VV ECLS

## Abstract

Lung volume reduction surgery (LVRS) represents an important treatment option in carefully selected patients with end-stage lung emphysema. The aim of this study was to assess the efficacy and safety of nonintubated LVRS compared to intubated LVRS in patients with preoperative hypercapnia and lung emphysema. Between April 2019 and February 2021, n = 92 patients with end-stage lung emphysema and preoperative hypercapnia undergoing unilateral video-assisted thoracoscopic LVRS (VATS-LVRS) performed in epidural anesthesia and mild sedation (nonintubated, group 1) or conventional general anesthesia (intubated, control, group 2) were prospectively enrolled in this study. Data were retrospectively analyzed. In all patients, low-flow veno-venous extracorporeal lung support (low-flow VV ECLS) was applied as a bridge through LVRS. Ninety-day mortality was considered as the primary outcome. Secondary endpoints included: chest tube duration, hospital stay, intubation and conversion to general anesthesia. Intergroup analysis showed no significant difference between the baseline data and patients’ demographics. N = 36 patients underwent nonintubated surgery. VATS-LVRS under general anesthesia was performed in n = 56 patients. The mean duration of postoperative VV ECLS support was 3 ± 1 day in group 1 compared to 4 ± 1 in group 2. The 90-day mortality rate was 3% in group 1 compared to 7% in group 2. In group 1, all chest tubes were removed 5 ± 1 day (range 4–32 days) and 8 ± 1 day (range 4–44 days) in the control group after the surgery (*p* < 0.02). Prolonged chest tube therapy (>8 days) was observed in n = 3 patients in group 1 and n = 11 patients in the control group. The mean ICU stay was 4 ± 1 days in group 1 compared to 8 ± 2 days in the control group (*p* = 0.04). The mean hospital stay was significantly shorter in the nonintubated group 1 (6 ± 2 days vs. 10 ± 4 days, *p* = 0.01). Conversion to general anesthesia was necessary in one patient due to severe pleural adhesions. Nonintubated VATS-LVRS in patients with end-stage lung emphysema and hypercapnia is effective and well tolerated. Compared to general anesthesia, a reduction in mortality, chest tube duration, ICU and hospital stay and lower rate of prolonged air leak was observed. VV ECLS increases intraoperative safety and mitigates postoperative complications in such “high-risk” patients.

## 1. Introduction

Nonintubated thoracic surgery (NITS) involves procedures performed under different regional anesthesia techniques in awake or mildly sedated, spontaneously breathing patients [1,2]. The purpose of this approach is to avoid adverse events related to mechanical ventilation under general anesthesia, to speed up recovery and to optimize perioperative outcomes. Moreover, a reduction in procedure-related side effects is one of the rationales behind this strategy [3]. Singular randomized studies demonstrated the efficacy of nonintubated video-assisted thoracoscopic surgery (NI-VATS) in the management of pleural diseases, minor and major anatomical lung resection for lung cancer [4,5,6] and even lung volume reduction surgery (LVRS) for severe emphysema [7]. Nevertheless, the mostly accepted indication for NI-VATS includes minor procedures which are technically easy to perform as well as surgical management of patients with significant risks for intubated general anesthesia. On the other hand, the utilization of this nonintubated approach in major procedures such as anatomic lung resections and LVRS is still controversial due to technical or lung functional challenges including, e.g., massive pulmonary hyperinflation and preoperative hypercapnia [3].

It has been evident for decades now that LVRS is an effective treatment tool for patients with end-stage lung emphysema and massive pulmonary hyperinflation. However, the benefit of this surgical approach is mainly reported in carefully selected patients [8,9]. Furthermore, patients with lung emphysema frequently present with persistent hypercapnia and those are at high risk for LVRS. Therefore, the perioperative management of those patients remains challenging [9]. In contrast, our previous and other studies have addressed the beneficial effects of LVRS in patients with preoperative hypercapnia [10,11]. There, we postulated that optimized perioperative management including low-flow veno-venous extracorporeal lung support (low-flow VV ECLS) enables such procedures in this high-risk patient cohort and this was underlined by our findings.

The aim of this current study was to assess the efficacy and safety of nonintubated LVRS compared to intubated LVRS in patients with preoperative hypercapnia and end-stage lung emphysema.

## 2. Patients and Methods

### 2.1. Ethical Statement

The authors have declared that this study was performed in accordance with research ethical guidelines. The study protocol was conducted in accordance with the ethical principles of the Declaration of Helsinki. This paper is exempt from ethical committee approval. Reason: this study is a retrospective analysis of routine clinical data with no information regarding personal data such as name, address or pictures. According to our local ethics committee (Ibbenbueren General Hospital), no ethical approval was required prior to this study. Informed consent for performing the therapy was taken from all patients if possible. If this was not possible, informed consent was taken from legal guardians prior to therapy.

### 2.2. Patients

Between April 2019 and February 2021, patients who presented with end-stage lung emphysema and hypercapnia that underwent LVRS were included into this analysis. Evaluation for surgery was carried out and the indication for surgery decided by the local interdisciplinary lung emphysema board. Data were collected prior to surgery at hospital admission and postoperatively at the day of discharge. Consent from patients for the LVRS procedure was obtained prior to surgery. Patients who consented to NI-VATS procedures were included into the nonintubated group 1. All other patients underwent LVRS with general anesthesia (group 2).

The standard preoperative institutional evaluation for LVRS included: 3D CT scan with volumetric quantification of the emphysematic target zones, ventilation/perfusion scintigraphy (V/Q) scanning, exercise capacity (6 minutes walking distance: 6 MWD and stair climbing), blood gas analysis (BGA), carotid duplex ultra-sonography, transthoracic echocardiography and myocardial scintigraphy as already described in our previous studies [11]. In case of inadequate or difficult echocardiographic evaluation, right heart catheterization was performed prior to surgery.

### 2.3. Inclusion Criteria

After completion of the evaluation process, patients were presented to the local interdisciplinary lung emphysema board. Patients presented with COPD Gold IV, heterogeneous emphysema and preoperative hypercapnia were included into this study. Hypercapnia was defined as an arterial carbon dioxide partial pressure (PaCO_2_) of >6 kPa (>45 mmHg).

### 2.4. Exclusion Criteria

Those were defined as age >80 years, presence of pulmonary arterial hypertension (pulmonary arterial systolic pressure ≥ 35 mmHg/ ≥ 4.6 kPa), presence of nicotine consumption, acute infectious exacerbation, evidence of homogeneous distribution of emphysema with absence of clear target zones and cardiac function impairment.

### 2.5. Selection Criteria for Nonintubated VATS-LVRS

Patients wanting a nonintubated approach were eligible for NI-VATS LVRS. In a first step, patients were informed about the nonintubated and the intubated approach. Thereafter, all patients received the opportunity to discuss all their related questions and then decided on their favored approach.

### 2.6. Contraindications for Nonintubated VATS-LVRS Were Defined as Following

- Body mass index >32 kg/m^2^;
- Expected difficult airway management;
- Contraindications for epidural anesthesia;
- Pre-existing cognitive impairment;
- Excessive coughing;
- Relevant cardiovascular comorbidities.

### 2.7. Definition of Outcomes

#### 2.7.1. Primary Outcomes

Ninety-day mortality was considered as the primary outcome of this study.

#### 2.7.2. Secondary Outcomes

Chest tube duration, hospital stay, intubation and conversion to general anesthesia were considered as secondary endpoints.

Furthermore, BGA was performed preoperatively, intraoperatively after utilization of veno-venous extracorporeal lung support (VV ECLS) during VATS-LVRS and postoperatively at least twice daily until the day of discharge to optimize parameters in case of noninvasive ventilation or high-flow oxygen supply. Additionally, this was necessary to estimate the need for bronchoscopy and assess the efficacy of respiratory therapy during the postoperative course. Pulmonary function testing, exercise capacity measured in stair/step climbing, 6-min walking distance (6 MWD), dyspnea scale (Borg scale) and health-related quality of life (verbal rating 1–10) were recorded preoperatively and postoperatively at the day of discharge.

### 2.8. Low-Flow VV ECLS

Based on our experience and recently published data, all patients presented with preoperative hypercapnia were perioperatively supported with low-flow veno-venous extracorporeal lung support (low-flow VV ECLS) to avoid intraoperative severe hypercapnia. VV ECLS was applied intraoperatively and continued postoperatively. Cannulation was performed using local anesthesia at the cannulation site. In all cases, VV ECLS was implemented via the right jugular vein using a 22 French (Fr.) Twin-Port double lumen cannula as previously described (NovaPort Twin^®^, Novalung, Heilbronn, Germany) [11]. ECLS was utilized using the iLA-activve system^®^ (Novalung, Heilbronn, Germany). For systemic anticoagulation, Argatroban was administered with a target activated partial thromboplastin time (aPTT) of 45–50 s. This was first started after surgery at the ICU.

### 2.9. Surgical Approach and Anesthesiologic Management

In all study patients, epidural anesthesia was applied before inserting ECLS and prior to surgery. This was usually placed at the T3–T5 level. The dosage of anesthetic administered was adjusted during the procedure. VATS-LVRS was performed using a uniportal approach in both groups.

For patients in the nonintubated group, 10 mL of lidocaine 2% was nebulized through an oxygen mask approximately 30 min prior to surgery, to minimize coughing during the procedure. Patients were mildly sedated with either propofol or dexmedetomidine (Dexdor^®^, Orion Corporation Orion Pharma Orionintie, Espoo, Finland). Oxygen was supplied via facial mask throughout the procedure. In case of hypoxia (BGA: PaO_2_ < 50 mmHg/ < 6.66 kPa), supplement oxygen was applied using nasal high flow. Patients remained spontaneously breathing and were responsive at all times during the procedure. For this purpose, bispectral index (BIS) was additionally used to monitor the depth of anesthesia with a value maintained between 70 to 80. Intercostal nerve block was applied percutaneously before the incision was made and intraoperatively under direct vision at the site of the incision as well as the intercostal space above and below. After incision of the pleura, the emphysema target zones were identified. If more deflation of the lung was necessary, the lung was lightly compressed by using a swab. In case of increased coughing during this maneuver, a vagal block was performed.

Type of resection and resection areas for both groups are summarized in Table 1.

### 2.10. Statistical Analysis

All sets of data were statistically analyzed and graphically presented using GraphPad Prism 9.0 (GraphPad Software, Boston, MA, USA). All variables were examined by an exploratory data analysis method and recorded descriptively. A normal distribution was tested using the Shapiro–Wilk test. For normally distributed data, independent sample *t*-test was used. Data are reported as mean ± standard deviation. A value of *p* < 0.05 was defined as the critical value (statistically significant).

## 3. Results

In group 1, 36 patients (n = 18 females) with a mean age of 64 ± 5 years (range 47–79 years) were included into the analysis. In group 2, 56 patients (n = 26 females) with a mean age of 63 ± 4 years (range 42–78 years) were identified. Outcomes for both groups are summarized in Table 2.

Regarding the mortality rate, we observed a lower 90-day mortality in group 1 (3%) compared to group 2 (7%). There was no significant difference in mortality between both groups (log rank test, *p* = 0.3; adjusted hazard ratio 0.37, 95% CI 0.06 to 2.2). During the early postoperative course, one patient died in the nonintubated group due to severe pneumogenic sepsis. Whereas, in group 2, n = 4 patients died due to severe pneumogenic sepsis (n = 2) and acute right heart failure (n = 2).

Conversion to mini-thoracotomy was necessary due to intraoperative severe adhesions in 1 patient in group 1 and 4 patients in group 2. No patient required postoperative reintubation for respiratory failure in both groups as a benefit of postoperative low-flow VV ECLS. The mean duration of postoperative ECLS was 3 ± 1 day (1–13 days) in group 1 compared to 4 ± 1 day (1–15 days) in group 2 (*p* = 0.6). Complications related to VV ECLS were not recorded. The mean ICU stay was 4 ± 1 days (1–20 days) in group 1 and 8 ± 2 (2–64 days) in group 2 (*p* = 0.04). In addition, the mean postoperative hospital stay was significantly shorter in group 1 (6 ± 2 days, range 4–33) than in group 2 (10 ± 4 days, range 3–77 days, *p* = 0.01).

In group 1, all chest tubes were removed 5 ± 1 day (range 4–32 days) and 8 ± 1 day (range 4–44 days) in the control group after the surgery (*p* = 0.02). Prolonged chest tube duration (>8 days) with prolonged air leakage was documented in n = 3 patients in the nonintubated group and n = 11 patients in the control group.

**Figure 1:**
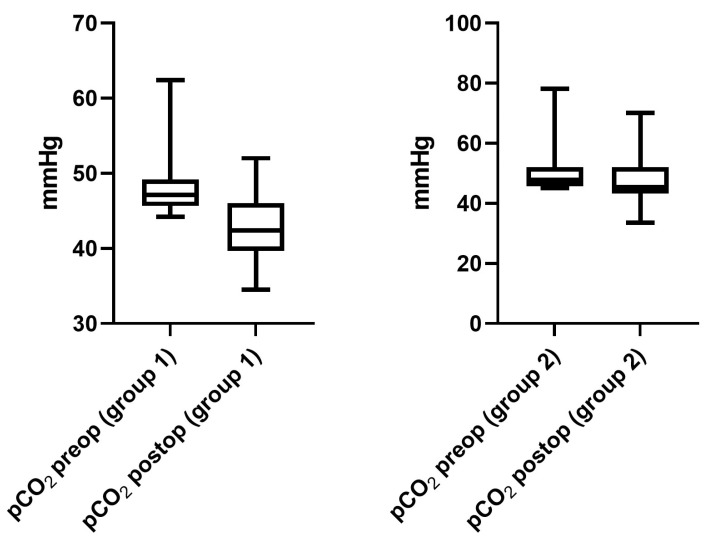
PaCO_2_ levels pre- and postoperatively (day of discharge) in both groups. In both groups, a significant decrease in PaCO_2_ levels was observed at the day of discharge compared to preoperative measures.

The mean preoperative PaCO_2_ level was 48.1 ± 1.4 mmHg (6.4 ± 0.18 kPa, range 45.1–62.4 mmHg/6.01–8.31 kPa) in group 1, compared to 51.1 ± 1.5 mmHg (6.81 ± 0.2 kPa) in group 2 (range 45.1–78.1 mmHg/6.01–10.4 kPa, *p* = 0.04). At the day of hospital discharge, a mean PaCO_2_ of 42.7 ± 1.7 (5.69 ± 0.22 kPa, range 34.5–52 mmHg/5.8–6.93 kPa) was recorded in group 1, which was significantly lower compared to preoperative measures (*p* < 0.0001). In group 2, the mean PaCO_2_ level at the day of discharge was 47.6 ± 2.4 mmHg (6.34 ± 0.32 kPa, range 33.5–70.1 mmHg, *p* = 0.01) compared to preoperative PaCO_2_ levels (Figure 1).

On preoperative lung function assessment in group 1, the mean forced expiratory volume in 1 second (FEV1) was 29.5 ± 2.8% of the predicted values (range 11–44%) compared to 29.5 ± 2.1% (range 19–45%) postoperatively (*p* = 0.9). The mean postoperative vital capacity (VC) was 53.3 ± 2.6% (range 37–94%) and 54.6 ± 3.6% of the predicted values prior to surgery (range 27–80%, *p* = 0.75).

In addition, in group 2, postoperative lung function measurements were similar to preoperative values. The mean FEV1 was 26.6 ± 2.2% (range 11–68%) prior to surgery compared to 28.1 ± 1.5% (range 16–60%) postoperatively (*p* = 0.6). The mean preoperative VC was 50.1 ± 3.4% (range 25–97%) versus 52.1 ± 4.3 postoperatively (range 26–78%, *p* = 0.6). In n = 2 patients in group 1 and n = 5 patients in group 2, lung function tests could not be performed due to rapid exhaustion during the examination and poor general physical condition.

**Figure 2:**
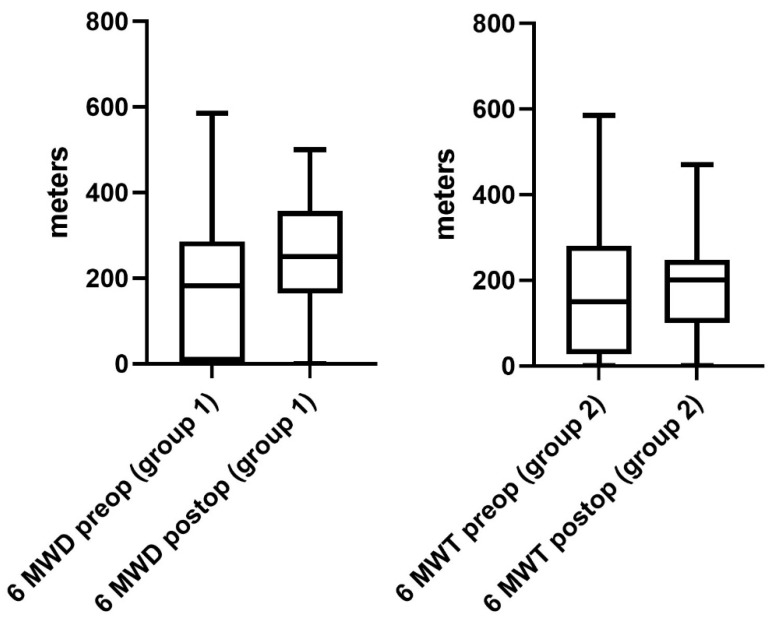
Performance status (6 MWD). Shows a significant improvement in the walked distance in 6 min in group 1 at the day of discharge. In addition, although not significant, a trend toward better performance compared to preoperative measurements was observed in group 2.

**Figure 3:**
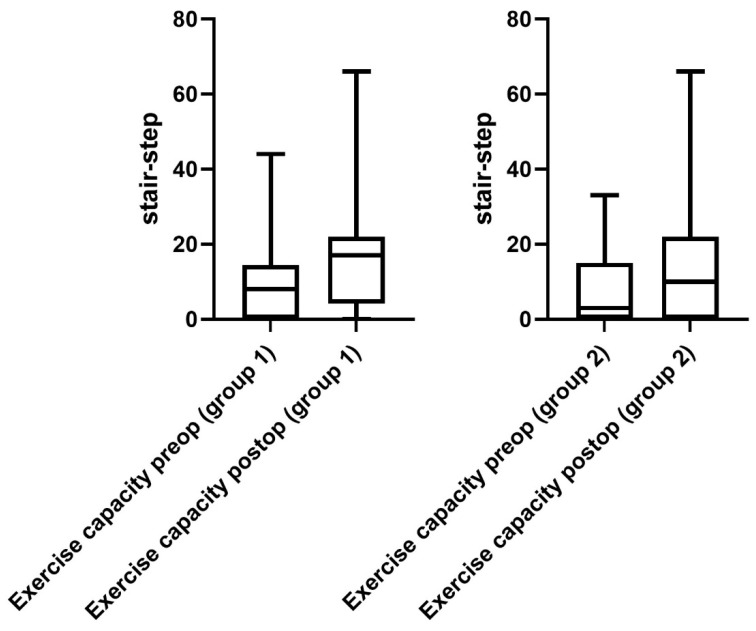
Performance status (exercise capacity). Exercise capacity measured in stair/step climbing improved significantly after surgery in both groups.

**Figure 4:**
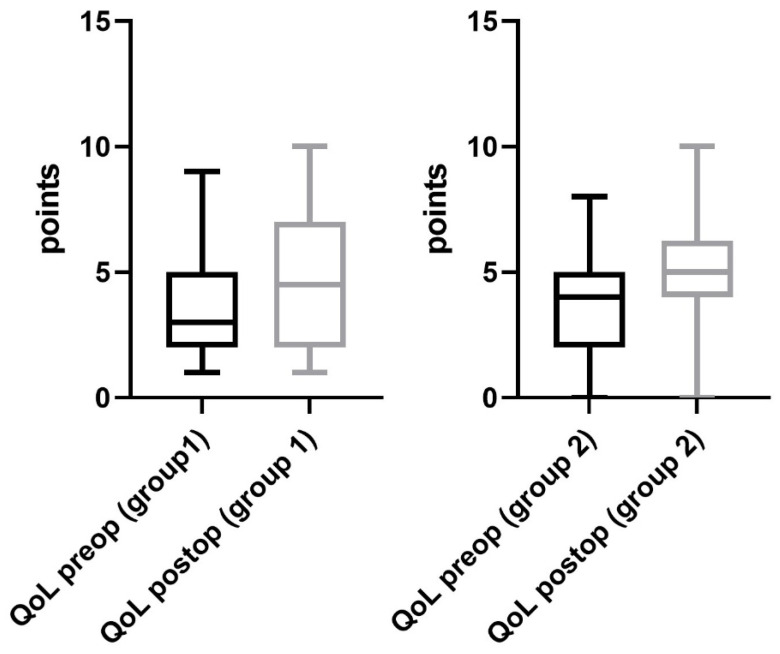
Quality of life. Quality of life (QoL) recorded in both groups improved significantly after surgery.

**Figure 5:**
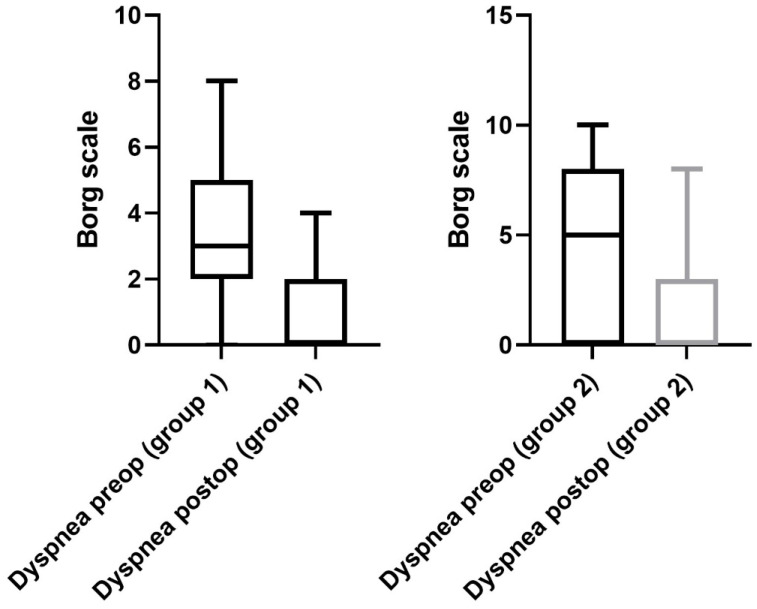
Dyspnea. A postoperative significant improvement was recorded regarding the dyspnea scale in both groups.

Regarding the performance status of patients in group 1, a significant improvement was observed after surgery. The 6 MWD improved significantly from 171 ± 18 m (range 0–585 m) to 256 ± 22 m (range 0–500 m) at the day of discharge (*p* = 0.02, Figure 2). Furthermore, the mean exercise capacity improved significantly from 9 ± 2 (range 0–44) to 19 ± 2 (0–66), (*p* = 0.01, Figure 3). In addition, a significant improvement in quality of life from 3 ± 1 (range 1–8) to 5 ± 1 points (range 0–10) was documented postoperatively (*p* = 0.03, Figure 4). The postoperative dyspnea score was 1 ± 1 (range 0–4) compared to 3 ± 1 (range 0–8) postoperatively (*p* < 0.0001, Figure 5).

Pre- and postoperative performance status measurements in group 2 revealed an increase in 6 MWD (mean preoperative 6 MWD of 170 ± 17, range 0–450 m vs. postoperative 6 MWD 186 ± 13, range 0–450 m, *p* = 0.5, Figure 2). Exercise capacity measurements showed a significant improvement postoperatively (preoperative stair/steps of 6 ± 2, range 0–33 vs. 12 ± 3, range 0–66 postoperatively, *p* = 0.01, Figure 3). In addition, a significant increase in quality of life (3 ± 1 preoperatively, range 0–8 vs. 5 ± 1 at the day of discharge, range 0–10, *p* = 0.0006, Figure 4) and dyspnea scale (4 ± 1 preoperatively, range 0–10, vs. 1 ± 1 postoperatively, range 0–8, *p* < 0.0001, Figure 5) were noticed in group 2.

## 4. Discussion

LVRS has been reported as a valuable treatment option for patients with severe lung emphysema, thereby improving lung function and physical performance. Nevertheless, careful patient selection and perioperative management may influence patient outcomes and reduce morbidity and mortality and patients at a high risk for LVRS are excluded from surgery in many programs. In this study, we retrospectively evaluated the feasibility and efficacy of LVRS in a nonintubated approach compared to conventional LVRS with general anesthesia. The main findings showed a reduction in the mortality rate as well as in ICU stay and postoperative hospital stay. The last finding is most likely due to a significantly shorter chest tube duration in the nonintubated group compared to patients with general anesthesia. The significant difference in performance status after surgery may suggest that patients after nonintubated LVRS recover faster and are able to engage in postoperative physical exercising programs earlier than patients in the control group.

Nonintubated thoracic surgery is not a novel invention. In the early 20th century, lung resection was performed on awake patients using various local anesthesiologic regimens including nerve blocks. Lobectomies and pneumonectomies in awake patients were reported as early as 1936 [12]. Over the years, different types of bronchial blocker systems and different types of endotracheal tubes have been developed. This development continued until the double-lumen endotracheal tube was introduced, which enabled single lung ventilation under general anesthesia. With the focus on enhanced recovery protocols and the development of minimally invasive thoracic surgical procedures, NI-VATS is nowadays a subject of renewed interest and is increasingly integrated in this concept [13,14,15]. In contrast to the awake procedures in the times of Sir Ivan Magill [12], the patient is not necessarily awake during the operation. Rather, the focus is on preserving spontaneous breathing.

In recent years, NI-VATS has gained popularity worldwide and already finds a place in thoracic surgery for minor procedures and even more complex procedures such as lobectomies and sleeve resections with promising results [5,6,16]. The main advantages of this technique can be clearly identified compared to thoracoscopic lung surgery with general anesthesia. For example, complications such as airway injury and ventilator-associated lung injury can be avoided [17]. Spontaneous breathing in NI-VATS enables favorable respiratory mechanics and mitigates the ventilation–perfusion mismatch during single-lung ventilation [18]. Furthermore, avoiding general anesthesia is reported to be beneficial in reducing morbidity rate, pneumonia and prolonged ventilator dependence as well as postoperative reintubation rate in COPD patients [19]. Recently, Jeon et al. investigated, in a randomized controlled study, the cytokine changes in patients undergoing thoracic surgery for lung cancer after an intubated and nonintubated approach. According to that study, a nonintubated approach may mitigate the inflammatory response after thoracic procedures for lung cancer compared to intubated surgery [20]. On the other side, caution is required with nonintubated strategies for patients with impaired lung function. This is related to the fact that prolonged spontaneous breathing could lead to hypoxia and hypercapnia. Moreover, conversion to general anesthesia could be immediately necessary and presupposes the presence of an experienced anesthesiologist. Hypoxemia, if present during nonintubated procedures, is mostly transient and may be easily avoided using nasal high-flow oxygen [21].

On the other side, patients with preoperative hypercapnia represent, in general, a patient cohort at “high risk” for LVRS [9,22,23]. These patients may intraoperatively develop high pCO_2_ levels during spontaneous breathing, especially during the pneumothorax situation, as well as during single-lung ventilation with general anesthesia. Consequently, respiratory acidosis, vasopressor requirement and unstable respiratory and hemodynamic conditions may occur, increasing the overall risk of the procedure [24,25]. Therefore, according to our recently reported experience, we applied VV ECLS in the cohort included in this study to ensure patient safety and avoid such complications, as previously described. Conversion to general anesthesia was only necessary for one patient due to strong adhesions but not because of hypercapnia. Intraoperative pCO_2_ values were maintained at normal levels in both groups, which was expected as a consequence of extracorporeal CO_2_ elimination, thus avoiding complications related to that. In our opinion, utilizing VV ECLS during NI-VATS LVRS in patients with preoperative hypercapnia is helpful to avoid massive intraoperative hypercapnia and perform such procedures.

Moreover, it is well known that patients undergoing LVRS for lung emphysema tend to develop postoperative prolonged air leak due to hyperinflation and the reduced tissue diameter of the lung. In addition, lung injury due to mechanical stress of the staple devices on lung tissue provokes a higher incidence for prolonged air leak. This has been reported to be one of the major complications influencing postoperative morbidity and mortality for patients undergoing LVRS. We have previously shown the effect of forced intraoperative mechanical ventilation on postoperative air leak. Therefore, avoiding mechanical ventilation and reventilation of the resected lung may help to minimize lung tissue injury and, consequently, to reduce air leak and chest tube duration [26,27]. In this context, Tacconi et al. reported the initial experience with nonresectional awake LVRS for patients with lung emphysema compared to LVRS under general anesthesia [7]. In that study, a significantly lower rate of prolonged air leak and, consequently, a shorter hospital stay was observed in the awake group. In our study, we also clearly observed a significant reduction in chest tube duration in the nonintubated group compared to the control group, which highlights a beneficial effect of the nonintubated approaches in emphysema patients. Values for postoperative air leakage in both groups were not considered to be an endpoint in this study and were unfortunately not documented. Therefore, this observation regarding shorter chest tube duration needs more investigation in further randomized controlled studies.

Despite the potential physiological and surgical benefits of NI-VATS, few studies to date have demonstrated a reduction in postoperative pulmonary complications. Randomized trials have reported a shorter time for anesthetization [28], reduced hospital stay and perioperative morbidity [3,5,7]. So far, the long-term effects of NI-VATS are not clear. To our knowledge, this is the first study demonstrating a nonintubated approach in patients with preoperative hypercapnia and end-stage lung emphysema undergoing VATS-LVRS. In order to reduce the perioperative risk, we implemented a well-established ECLS approach in this study cohort. Thus, we were able to reduce the mortality rate for patients undergoing nonintubated VATS-LVRS compared to general anesthesia. Additionally, a significantly shorter chest tube duration and a reduced hospital stay in the nonintubated group was documented. Significant improvement in physical condition and quality of life after surgery was achieved in both groups. However, the nonintubated LVRS patients tended to recover faster physically after surgery.

Finally, an experienced, routinized interdisciplinary team including surgeons and anesthesiologists plays an important role in guaranteeing success and safety during nonintubated approaches especially in those high-risk patients considered for LVRS. Despite the promising results of this study, some limitations should be addressed. The cohort included is small and represents our single center experience. Further studies on larger cohorts are needed to verify our positive results. In addition, further follow-up is necessary to evaluate the long-term effects of this promising approach.

## Figures and Tables

**Table 1:** Type of lung resection in both groups.

Type of Resection	No. of Patients (%) (Group 1, n = 36)	No. of Patients (%) (Group 2, n = 56)
**Lobar resection**	20 (56)	28 (50)
**Sublobar resection** **S6-sparing** **LUL S1–S3**	2 (6) 0 (0) 2 (100)	5 (9) 2 (40) 3 (60)
**Apical wedge**	14 (38)	23 (41)

S6-sparing: Segment 6 sparing resection of the lower lobe; LUL S1–S3: left upper lobe trisegmentectomy.

**Table 2:** Patient outcome measures.

Characteristic	Group 1 (n = 36)	Group 2 (n = 56)	*p*-Value
**Age (years)**	64 ± 5 [47–79]	63 ± 4 [42–78]	n.s.
**Sex (%)**			
**Female**	18 (50)	26 (46.4)	n.s.
**Male**	18 (50)	30 (53.6)	n.s.
**ICU stay (days)**	4 ± 1 [1–20]	8 ± 2 [2–64]	0.04
**Postop. hospital stay (days)**	6 ± 2 [4–33]	10 ± 4 [3–77]	0.01
**Chest tube duration (days)**	5 ± 1 [4–32]	8 ± 1 [4–44]	0.02
**VV ECLS duration (days)**	3 ± 1 [1–13]	4 ± 1 [1–15]	0.6

## Data Availability

Data are available on request. The data underlying this article will be shared on reasonable request to the corresponding author.

## Abbreviations

VV ECLS	Veno-venous extracorporeal lung support.
NITS	Nonintubated thoracic surgery.
NI-VATS	Nonintubated video-assisted thoracoscopic surgery.
LVRS	Lung volume reduction surgery.
6 MWD	Six minutes walking distance.
BGA	Blood gas analysis.
PaCO_2_	Carbon dioxide partial pressure.
BIS	Bispectral index.
